# Bi-clustering of metabolic data using matrix factorization tools

**DOI:** 10.1016/j.ymeth.2018.02.004

**Published:** 2018-12-01

**Authors:** Quan Gu, Kirill Veselkov

**Affiliations:** a MRC-University of Glasgow Centre for Virus Research, University of Glasgow, Garscube Estate, Glasgow G61 1QH, UK; b Department of Surgery and Cancer, Faculty of Medicine, Imperial College London, Sir Alexander Fleming Building, Exhibition Road, South Kensington, London SW7 2AZ, UK

**Keywords:** Bi-clustering, Matrix factorization, Bi-cross validation, Metabolic data

## Abstract

Metabolic phenotyping technologies based on Nuclear Magnetic Spectroscopy (NMR) and Mass Spectrometry (MS) generate vast amounts of unrefined data from biological samples. Clustering strategies are frequently employed to provide insight into patterns of relationships between samples and metabolites. Here, we propose the use of a non-negative matrix factorization driven bi-clustering strategy for metabolic phenotyping data in order to discover subsets of interrelated metabolites that exhibit similar behaviour across subsets of samples. The proposed strategy incorporates bi-cross validation and statistical segmentation techniques to automatically determine the number and structure of bi-clusters. This alternative approach is in contrast to the widely used conventional clustering approaches that incorporate all molecular peaks for clustering in metabolic studies and require *a priori* specification of the number of clusters. We perform the comparative analysis of the proposed strategy with other bi-clustering approaches, which were developed in the context of genomics and transcriptomics research. We demonstrate the superior performance of the proposed bi-clustering strategy on both simulated (NMR) and real (MS) bacterial metabolic data.

## Highlights

• We propose a positive matrix factorization bi-clustering strategy for metabolic data.
• The approach automatically determines the number and composition of bi-clusters.
• We demonstrate its superior performance compared to other techniques.

## 1 Introduction

Modern Nuclear Magnetic Resonance (NMR) spectroscopy and Mass Spectrometry (MS) technologies generate vast amounts of unrefined metabolic data in biomedical studies [1], [2]. The metabolic signature of a complex biological mixture (‘metabolic profile’), such as that obtained from analysis of biofluids, consists of overlapping signals of hundreds to thousands of distinct chemical entities influenced by genes, treatment, gut microbiota and other environmental factors. This myriad of factorial influences results in complex inter-relationships between both spectral observations and variables. The clustering and related unsupervised learning tools are frequently used to discover patterns of relationships between samples and metabolites [3], [4].

Given a two-dimensional data matrix X with m rows (samples) and n columns (variables), traditional clustering analysis aims to identify groups of samples (or respectively variables) that exhibit similar behaviour across all variables (or respectively samples). This strategy is useful to perform global partitioning of the data matrix. In “-omics” studies, molecules (e.g., genes or metabolites) can be involved in one or more biological processes and exhibit similar patterns of behaviour across a subset of samples (but not necessarily all). The bi-clustering strategies are more suitable in such cases. The objective of biclustering is to perform simultaneous clustering of both rows and columns in the data matrix [5]. This means that clustering derives a global model, while biclustering produces a local model. Each row in a bicluster is selected using only a subset of the columns and each column in a bicluster is selected using only a subset of the rows.

In “omics” sciences, the (bi)clustering methods have been widely applied to gene expression data matrix, where rows represent gene transcripts and column represent conditions/samples. The data matrix element corresponds to the expression level of a gene under a specific condition [6]. Unlike clustering algorithms, the goal of the technique is to identify groups of genes that show similar activity patterns under a specific subset of the experimental conditions. Such biclusters are biologically relevant since they not only capture the correlated genes but also identify the genes that do not behave similarly in all conditions [7]. Thus, the biclustering algorithms have been shown to discover more biologically relevant clusters, compared to conventional global clustering techniques.

The biological application of biclustering algorithm was first used by Cheng and Church (CC) [8] for gene expression data. After this initial approach, a number of biclustering algorithms including Spectral [9], Plaid [10], BiMax [11], Xmotifs [12], OPSM [13], ISA [14], QUBIC [15] and FABIA [16] have been developed to identify various types of biclusters for gene expression data. A useful criterion to evaluate a biclustering algorithm is based on the identification of the type of biclusters the algorithm is able to find. In broad terms, the types of biclusters can be divided into [5]:

1. Biclusters with constant values.
2. Biclusters with constant values on rows or columns.
3. Biclusters with coherent values (addictive model).
4. Biclusters with coherent values (multiplicative model).
5. Biclusters with coherent evolutions.

The spectroscopic data (NMR or MS), with rows representing the samples and columns representing variables, can be considered as a linear combination of metabolite peaks plus noise, which is corresponding to the biclusters with constant values on columns or overlapped. However, the aforementioned biclustering methods with low anti-disturbing and fault tolerance are only suitable for gene expression data; in this scenario, the rows (genes) and columns (conditions) are fixed without overlapping partitions of conditions from the experiment.

Matrix factorization is a decomposition of a data matrix into a product of matrices, either for regularization or for interpretation. A variety of matrix factorization methods by incorporating different constraints, e.g. singular value decomposition (SVD) [17], principal component analysis (PCA) [18], and non-negative matrix factorization (NMF) [19], could be applied to minimise the dimensionality of the data yielding a representation of conditions as a linear combination of a reduced set of factors [20]. The factor scores/loadings represent sets of rows or columns that behave in a strongly correlated manner with the original data. In the gene expression data, various matrix factorization methods have been used to cluster genes or conditions based on local patterns and predict functional relationships [21], [22]. Apart from the genomic datasets, the matrix factorization tools could also be used for exploring the spectroscopic datasets for their output matrices represent the relevance of samples and compound variables simultaneously [23].

In this paper, we first present a biclustering technique based on matrix factorization to identify subsets of correlated metabolites exhibiting similar patterns of behaviour across a subset of samples (but not necessarily all). The critical factor is how to select the number of biclusters. We have thus incorporated the bi-cross validation and statistical segmentation techniques to automatically determine the number and structure of bi-clusters. This alternative approach is in contrast to the widely used conventional clustering approaches that use all molecular peaks for clustering in metabolic studies and require *a priori* specification of the number of clusters. We perform the comparative analysis of the proposed strategy with other bi-clustering approaches, which were developed in the context of genomics and transcriptomics research.

## 2 Materials and methods

### 2.1 Synthetic dataset

A bicluster is a subset of rows that exhibit similar behaviour across a subset of columns, and vice versa. Given a data matrix *A*, a bicluster *A_IJ_*(*I*,*J*) denotes the submatrix of *A* that contains only the elements $a_{\mathrm{ij}}$ belonging to the submatrix with a set of rows *I* and set of columns *J*.

**Fig. 1:**
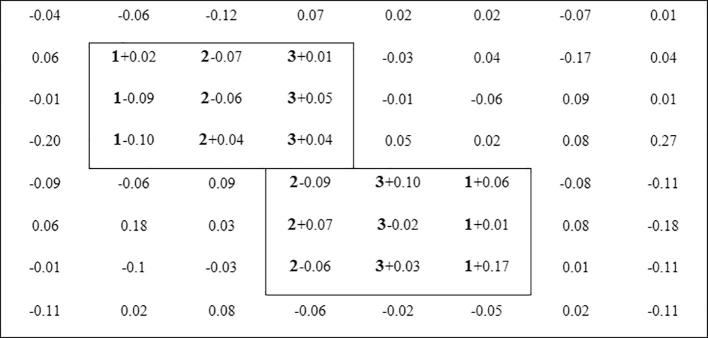
A simple example of simulation data matrix with biclusters of constant values on columns.

There are two ways to generate synthetic NMR/MS datasets for evaluating the algorithm performance of the metabolites biclustering. Considering the characteristic of NMR/MS spectroscopic data matrix, the first way is to use biclusters with constant values on columns to simulate the compound. Given the *K* bicluster, the dataset of *i* rows and *j* columns can be built by the equation

(1) $$X_{\mathrm{ij}}=\mu +\overset{K}{\underset{k=1}{\sum }}\beta_{\mathrm{jk}}\rho_{\mathrm{ij}}\kappa_{\mathrm{ij}}+\varepsilon_{\mathrm{ij}}$$

where $X_{\mathrm{ij}}$ represents the element in the bicluster, μ is the typical value and $\beta_{\mathrm{jk}}$ is the value for column *j* within the within the *k*-th bicluster. ρ and κ are indicator variables for row *i* and column *j* membership in the bicluster *k*. The noise ε is generated from a Gaussian distribution with zero mean and a varying standard deviation, i.e. ε∈N(0,δ), where δ is the noise level. Fig. 1 provides a simple example of 8 × 8 matrix with β = {1,2,3}. The typical value μ is set to 0 and the noise level δ is set to 0.1.

Aside from the method mentioned above, the MetAssimulo [24] is an important tool to simulate ^1^H NMR spectra of metabolic profiles. MetAssimulo is a package, which can create realistic metabolic profiles containing large numbers of metabolites with a range of user-defined properties based on the concentration information input by the user or constructed automatically from the Human Metabolome Database. For instance, if the concentration information is in the custom mode, the user could set the concentration information of ‘case’ samples by defining the fold-change of the mean and standard deviation of corresponding concentration in ‘control’ samples, which is constructed automatically from the Human Metabolome Database. Furthermore, MetAssimulo is able to simulate shifts in NMR peak positions that result from matrix effects (e.g., pH variation), which are often observed in metabolic NMR spectra.

In detail, the schema of building ^1^H NMR synthetic data by MetAssimulo is listed as follows:

**Step1:** Given the number of biclusters K ⊂ {1,2,…,*k*}, the number of samples in each biclusters M ⊂ {*m_1_*,*m_2_*,…,*m_k_*}, the number of metabolites in each biclusters N ⊂ {*n_1_*,*n_2_*,…,*n_k_*}.

**Step2:** For k∈K, m∈M and n∈N: set the fold-change of mean and standard deviation of the concentration of *n_k_* –th metabolite in *m_k_* ‘case’ samples.

**Step3:** Run the MetAssimulo package and simulate ^1^H NMR spectra of metabolic profiles, get the data matrix *A* and the median of the data matrix *A*_1/2_.

**Step4:** Align and normalize the data matrix.

**Step5:** Logarithm transfers the data matrix $A^{\prime }=\left|\frac{A}{A_{1/2}}\right|$ for biclustering analysis.

#### 2.1.1 Non-negative matrix factorization

Non-negative matrix factorization, also known as the classical NMF model is a useful algorithm in multivariate analysis and linear algebra, which has been successfully applied in chemometrics [19]. The technique can be applied to the analysis of multidimensional datasets to reduce the dimensionality, discover latent patterns and aid in the interpretation of the data.

The main difference between NMF and other classical factorization techniques such as SVD [17] and PCA [18] depends on the non-negativity constraints imposed on both score and loading vectors. In this way, output matrices can be interpreted as parts of the data or as subsets of elements that tend to occur together in sub-portions of the dataset [20]. Thus, the factor matrices produced by NMF (i.e., factor scores and factor loadings) that lend themselves to a relatively easy contextual interpretation, while the factor matrices obtained by the other classical factorization approaches, allow themselves to be of the arbitrary sign with no obvious contextual meaning.

The NMF algorithm is described as follows:

(2) $$A_{m\times n}=W_{m\times k}H_{k\times n}=\overset{k}{\underset{a=1}{\sum }}W_{m\times a}H_{a\times n}$$

where *A* is the positive data matrix with *m* samples and *n* variables, *k* is the number of components with *k* << min(*m*,*n*).

A solution to the NMF problem can be obtained by solving the following optimization object function:

(3) $$\underset{W\text{,}H}{\min}f(W\text{,}H)=\frac{1}{2}{\left\|A-\mathrm{WH}\right\|}_{F}^{2}$$

where *W* is a basis matrix, *H* is a coefficient matrix, ${\left\|\cdot \right\|}_{F}$ is the Frobenius norm and *W*, *H* ≥ 0 means that all elements of *W* and *H* are non-negative.

Considering the non-negativity of metabolites concentration in NMR/MS spectra, we use the NMF method to find biclusters of metabolites from NMR/MS spectra. As shown in the Eq. (2), under the number of biclusters *k*, the classical NMF approximately reproduce a ^1^H NMR spectroscopic data matrix *A* of dimension *m* samples and *n* variables as a product of two non-negative constraint matrices *W* and *H*. The *W* factor scores matrix has the dimension of a single array (*m* samples) and *k* biclusters, while the columns of factor loading matrix *H* are known as variable vectors and are in one-to-one correspondence with the NMR/MS spectra data matrix *A*.

#### 2.1.2 Bi-cross-validation

Given a large dataset matrix *A* of dimension m×n, several useful methods handle it to produce two matrices *W* and *H*, and the cross-validation(CV) is a practical algorithm to determine the number of rank of *W* and *H*, i.e. the number of component in the large matrix [17]. However, the result is affected by the noise of matrix and there is a risk of overfitting. Considering the noise and complexity in spectroscopic data and the overlap between metabolites, the cross-validation is not suitable for predicting the number of biclusters in these data matrices.

As illustrated by Owen and Perry [17], bi-cross-validation algorithm (BCV) is a useful tool that is generally applicable to outer product approximations, just as CV is for independent and identically distributed random variables sampling. The performance and robustness of BCV is better than CV, and is more suitable than CV for the unsupervised learning (e.g. matrix factorization).

In the present study, we use BCV of matrix factorization to predict the number of the biclusters. The schema of algorithm is listed as follows:

**Step1:** Given a data matrix $A\in {\left[0\text{,}\infty \right)}^{m\times n}$, row and column holdout subset I_l_ = {1,2, ,m}, J_l_ = {1,2,…,n}, for number of holdout *l =* 1,2,…,*L*, and list of ranks the number of metabolites in each biclusters *K* = {1,2,…,min(*m*,*n*)}.

**Step2:** For k∈K:BCV(k)←0

**Step3:** For l∈{1,2,…,L} and k∈K:$I\leftarrow I_{l}$, and $J\leftarrow J_{l}$, fit the matrix factorization model: $A_{-I\text{,}-J}≐W_{-I\text{,}-J}^{\left(k\right)}H_{-I\text{,}-J}^{\left(k\right)}$.

**Step4:** Reconstruct the matrix and get the confirming residual matrix ${\left\|A_{I\text{,}J}-A_{I\text{,}J}^{*(k)}\right\|}_{F}^{2}$, where $A_{I\text{,}J}^{*(k)}\leftarrow A_{I\text{,}-J}{\left(H_{-I\text{,}-J}^{\left(k\right)}W_{-I\text{,}-J}^{\left(k\right)}\right)}^{+}A_{-I\text{,}J}$.

**Step5:** Update the $\mathrm{BCV}(k)\leftarrow \mathrm{BCV}(k)+{\left\|A_{I\text{,}J}-A_{I\text{,}J}^{*(k)}\right\|}_{F}^{2}$.

#### 2.1.3 Other methodologies

Apart from matrix factorization, a myriad of bicluster techniques have been proposed for gene expression data [5], [11]. In this paper, the spectroscopic data (NMR or MS), rows represent the samples while columns represent variables, can be considered as a linear combination of metabolite peaks plus noise, which is corresponding to the biclusters with constant values on columns. For this reason, we compared our methods with the other biclustering techniques (e.g. Spectral [9], Plaid [10], BiMax [11], Xmotifs [12], ISA [14] and FABIA [16]) aimed at identifying constant columns.

Among these methods, the algorithms required for rescaling or iteration (e.g., Spectral and ISA) have longer running time on large datasets; the algorithms with methodology based on the binary value (e.g., BiMax, Xmotifs) are more sensitive to the noise of the data. Besides ISA, the performance of these methods is likely affected by the overlap of biclusters. Furthermore, Plaid, ISA and FABIA are also suitable for other bicluster classes. Brief methodological overview and the references of these biclustering techniques are listed in the Table 1.

**Table 1:** The summary of methods for identifying biclusters with constant row or column on the gene expression datasets.

Algorithm	Methodology	Description
BiMax [11]	Seeks the rectangles of ‘1’′s in a binary matrix	Only suitable for the bicluster with constant up-regulated condition; sensitive to the noise and number of biclusters; affected by the overlap
Plaid [10]	Assume the bicluster is generated as the sum of a background effect, cluster effects, row effects, column effects and random noise	Both suitable for conditions of the bicluster with constant value and constant row/column; sensitive to the noise; affected by the overlap
Spectral [9]	Advantages over SVD spectral analysis of the original or rescaling raw data	Both suitable for conditions of the bicluster with constant up- or down- regulated condition; not sensitive to the noise; not suitable for the discrete datasets; limited in running speed on large datasets; affected by the overlap
Xmotifs [12]	A nondeterministic greedy algorithm that seeks biclusters with conserved rows/columns	Only suitable for conditions of the bicluster with constant row/column; required for dataset discretized and more sensitive to the noise; affected by the overlap limited in running speed on large datasets; affected by the overlap
FABIA [16]	Analysis for bicluster acquisition models the data matrix as the sum of biclusters plus additive noise, bicluster is the outer product of two sparse vectors	Both suitable for conditions of the bicluster with constant value and constant row/column; not sensitive to the noise and the number of biclusters; affected by the overlap
ISA [14]	A nondeterministic greedy algorithm that seeks biclusters from starting with a seed bicluster and re-running the iteration steps	Both suitable for conditions of the bicluster with constant value and constant row/column; not sensitive to the noise the number of biclusters, and the overlaps; limited in running speed on large datasets

#### 2.1.4 Evaluating measurement

In this paper, we calculated the bicluster match score to evaluate the performance of biclsutering method, which was proposed by Prelic et al [11]. Without loss of generality, assume that s assigns larger scores to similar biclusters and smaller scores to dissimilar ones. $M_{1}\text{,}M_{2}$ are two sets of biclusters and the bicluster match score of sample $S_{s}$ can be calculated by the following equation:

(4) $$S_{s}(M_{1}\text{,}M_{2})=\frac{1}{\left|M_{1}\right|}\underset{b_{1}\in M_{1}}{\sum }\underset{b_{2}\in M_{2}}{\max}\left(\frac{\left|b_{1}\cap b_{2}\right|}{\left|b_{1}\cup b_{2}\right|}\right)$$

where *b*_1_, *b*_2_ are biclusters in the set $M_{1}\text{,}M_{2}$ respectively, $\left|b_{1}\cap b_{2}\right|$ is the number of data elements in their intersection, and $\left|b_{1}\cup b_{2}\right|$ is the number in their union. Similarity, the bicluster match score of variable *S_v_* can be calculated as well. The overall bicluster match score can be defined as $S\left(M_{1}\text{,}M_{2}\right)=\sqrt{S_{s}(M_{1}\text{,}M_{2})\cdot S_{v}(M_{1}\text{,}M_{2})}$.

Let *E* denote the ground truth bicluster set and *F* denote the set of found biclusters, the recovery score is *S*(*E*, *F*) and relevance score is *S*(*F*, *E*). If the recovery score *S* is maximized, it represents that the algorithm is found in all the expected biclusters. Similarly, if the relevance score *S* is maximized, all the found biclusters were expected.

In this study, we develop a visualization graphical user interface and work on the spectroscopic dataset. The graphical user interface written in Matlab is available by contacting the corresponding author.

## 3 Results and discussion

### 3.1 Evaluation of biclustering on the synthetic dataset

**Fig. 2:**
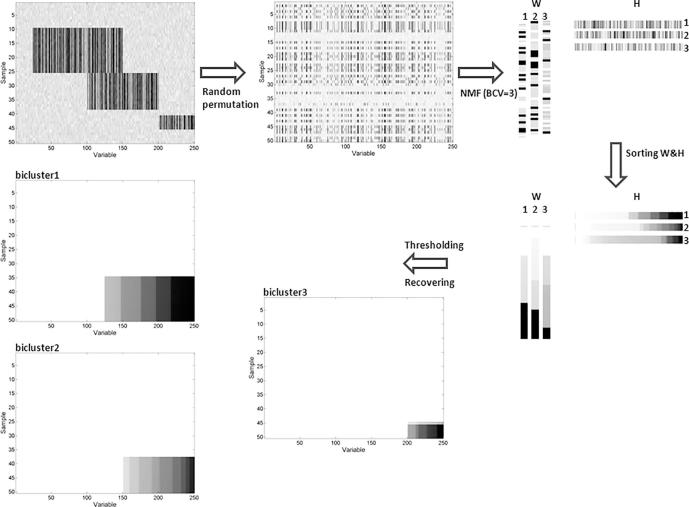
General schema of the method NMF approximates the synthetic data 1(noise level δ = 0.25) as a product of two submatrices, W and H. BCV is used for predicted the number of biclusters and thresholding algorithm is used for the identifying the indicator of each bicluster.

We explored the bicluster model on the synthetic datasets. Firstly, we built a synthetic dataset with only 50 samples and 250 variables for observing the performance of biclustering methods. From the Eq. (1), we set the number of biclsuter *k* = 3 with two of them having overlap. The value in each bilcuster is set as β∈{1,2,…,5}, the typical value is set as the $\mu =\min(|X_{\mathrm{ij}}|)$ meanwhile the noise level is set as δ∈[0,1]. Considering the randomness of the realistic datasets from experiment, we randomly permuted the samples (rows) and variables (columns) of the dataset and generated the synthetic dataset 1 (Fig. 2). The heatmap of the original dataset before rows and columns randomly permutated with the noise level δ=0.25. After the permutation of the rows and columns of the synthetic dataset, we calculated the average bicluster match scores to evaluate the performance of different biclustering models. The scheme of NMF model is shown in Fig. 2.

We also observed the performance of the bicluster algorithm on a synthetic dataset (synthetic data 2) with 30 samples generated by MetAssimulo. MetAssimulo can create realistic metabolic profiles containing large numbers of metabolites with a range of user-defined properties based on the concentration information input by the user or constructed automatically from the Human Metabolome Database. In the present study, the overall number of metabolites is 48, the number of biclusters is set to 3, and the fold-change values of mean and standard deviation of the concentration of metabolites are either positive or negative. Table 2 provides the metabolites implanted of each bicluster. Importantly, considering the peak shift of the output of MetAssimulo, the spectroscopic data is required for alignment and normalization. Following the recursive segment-wise peak alignment model [18] and logarithm transfer, we generated the synthetic dataset 2. As shown in the Fig. S1 (supplement file), the NMF model selects the correct ID of samples and hippuric acid (positive fold change of mean NMR spectra) within the bicluster 1 from the chemical shifts ^1^H ppm 7.50–7.90.

**Table 2:** Metabolites implanted of each bicluster in synthetic data 2 (the metabolites have the negative fold-change are in bold).

ID of bicluster	Metabolites in the bicluster
1	Citric acid, Creatine, Succinic acid, Hippuric acid, Serine
2	Creatinine, Citric acid, Glycine, **Formic acid**, Trimethylamine, **Hippuric acid**
3	**Creatinine, Formic acid**, Taurine, Betaine, **Guanidoacetic acid**, Trimethylamine

Apart from our two synthetic datasets, we also used the simulation dataset generated for gene expression by Eren et al. [7] to validate the characteristics of the bicluster models. The matrix contains 500 genes (rows), 200 conditions (columns), and each bicluster with 50 rows and 50 without the overlap.

In this paper, we separately tested the performance of six classical biclustering models, i.e. Spectral [9], Plaid [10], BiMax [11], Xmotifs [12], ISA [14] and FABIA [16], and three matrix factorization algorithms, i.e. PCA, NMF and sparse NMF(SNMF) [25]. In matrix factorization algorithms, the number of biclusters could be predicted by BCV. Additionally, since the matrix factorization algorithms could only generate score and loading matrices, thresholding algorithms are utilized for selecting the samples and variables in each biclusters.

**Fig. 3:**
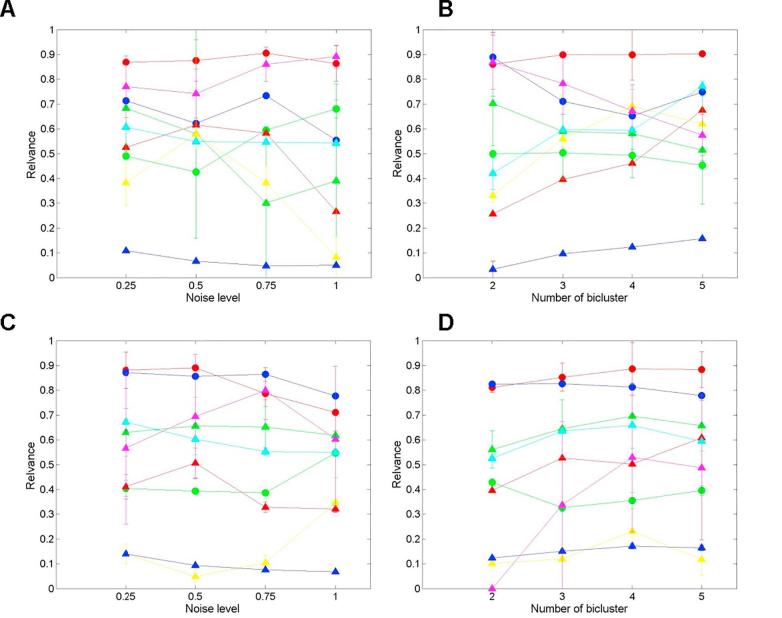
The bicluster model experiment on the synthetic datasets. (A) The relevance score of bicluster methods on synthetic gene expression data [7] with different noise level; (B) The relevance score of bicluster methods on synthetic gene expression data [7] of the different number of biclusters; (C) The relevance score of bicluster methods on synthetic spectroscopic data 1 (Fig. 2) with different noise level; (D) The average relevance score of bicluster methods on synthetic spectroscopic data 1 (Fig. 2) the different number of biclusters. The methods: NMF (red circle), PCA (blue circle), Sparse NMF(green circle), BiMax(red triangle), Plaid(green triangle), Spectral(yellow triangle), Xmotifs(blue triangle), Fabia(magenta triangle) and ISA(cyan triangle. (For interpretation of the references to colour in this figure legend, the reader is referred to the web version of this article.)

Fig. 3 summarises the relevance scores as a function of the number of biclusters and varying noise levels of the simulated datasets. We only represent the average relevance scores comparison but not the recovery score due to the similarity of both scores generated.

As expected, compared with six classical biclustering models, the matrix factorization methods have better robustness on the number of biclusters. It also validates the effectiveness of BCV prediction on the number of biclusters, which is an essential prerequisite for high quality performance. We tested the performance of six classical biclustering models and three matrix factorization algorithms on the simulation dataset of gene expression. The results of the average relevance scores for each bicluster model with different noise level and the number of biclusters are separately shown in the Fig. 3A and B. NMF achieves the highest relevance scores among the models, which validates the effectiveness of NMF model to identify the biclusters on the gene expression data. The methods Xmotif has the poorest performance on this dataset. With regards to the other methods, the performance of Plaid and FABIA is challenging as the number increased (Fig. 3B), whilst ISA and Spectral are negatively affected by the noise (Fig. 3A). However, as shown in the Figures, the SNMF is more sensitive to the noise than NMF. The reason is that the biclusters feature selection in sparse NMF is not based on the thresholding algorithm but on the sparseness of loading or score values, which is not stable in case of the overlap of the biclusters.

On the synthetic dataset 1 of spectroscopic data, we tested the performance of the present nine algorithms as well. The plots of the average relevance scores for each bicluster model with different noise level and the number of biclusters are separately given in the Fig. 3C and D.

As indicated in the Fig. 3C and D, the overall bicluster match scores of NMF and PCA are obviously highest among all the models. It indicates that the inherent clustering property of matrix factorization is more suitable for the spectroscopic data. As shown in the plot, the algorithms that seek local patterns (e.g., Xmotifs) are more sensitive to the noise, while the algorithms which fit a model of the entire dataset (e.g., ISA, Plaid) are less sensitive. Bimax, which is required for pre-processing of the data matrix into binary, has poor performance although it is effective on the gene expression synthetic datasets. In general, NMF has the best performance among all the algorithms on both synthetic datasets.

### 3.2 Evaluation of biclustering on the biological dataset

**Fig. 4:**
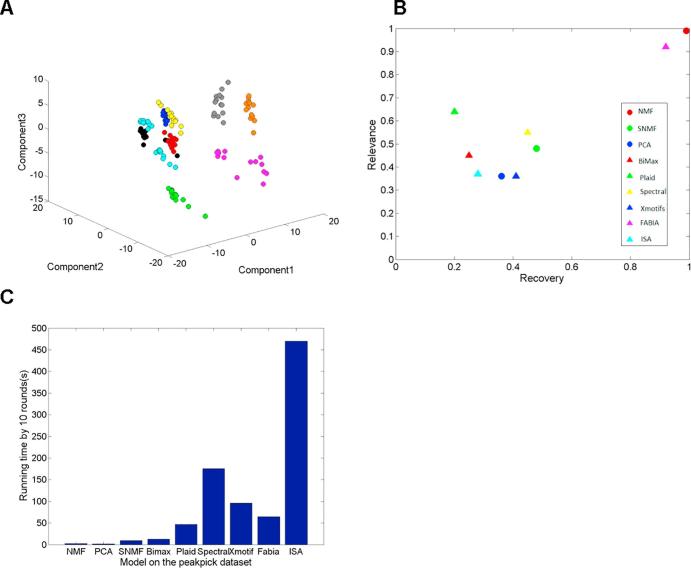
The bicluster model experiment on the bacteria MS spectra[29]. (A) The 3-D PCA score plot of dataset. The species: *C. koseri* (red), *K. pneumonia* (green), *P. mirabills* (blue), *S. aureus* (magenta), *S. pyogenes* (yellow), *E. coli* (cyan), *P. aeruginosa* (black), *S. agalactiae* (brown), *S. marcescens* (orange); (B) The average recovery and relevance bicluster match score of different bicluster methods. The methods: NMF (red circle), PCA (blue circle), Sparse NMF(green circle), BiMax(red triangle), Plaid(green triangle), Spectral(yellow triangle), Xmotifs(blue triangle), Fabia(magenta triangle) and ISA(cyan triangle); (C) The running time of the algorithms running by 10 rounds on the peakpick dataset. (For interpretation of the references to colour in this figure legend, the reader is referred to the web version of this article.)

**Fig. 5:**
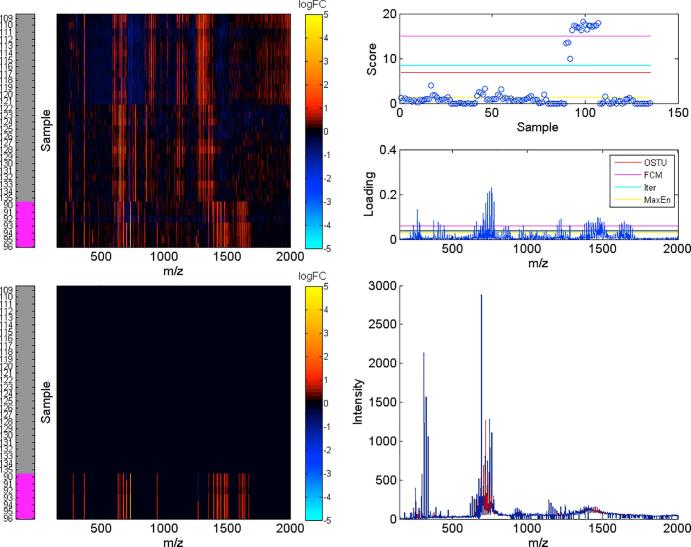
The biclustering of bacteria MS peak-picked dataset using NMF: The right-bottom subplot shows the median of the MS spectra. The variables (right-bottom) associated with samples within the selected bicluster are separately marked with red. The left-top section of the plot indicates the log transform of fold change according to mean of the MS spectra. The left-bottom subplot represents the recovering of selected bicluster. The samples within the selected bicluster are marked with the magenta in the left colour bar. The right-top section of the plot represents the score and loading of the dataset and the comparison of thresholding algorithms: OTSU(red), FCM(magenta), Iter(cyan) and MaxEn(yellow). The horizontal axis (i.e. *m*/*z* ratio) of the subplot keeps in consistent with each other when selecting the compounds. (For interpretation of the references to colour in this figure legend, the reader is referred to the web version of this article.)

We also explored the bicluster model on the MS dataset (negative ion) of nine bacterial species [29], [30]. The clusters can be distinguished by the visualization of the first three principal components in the PCA (Fig. 4A) belonging to Gram-positive *Streptococcus spp*., *Staphylococcus aureus*, Gram-negative *Pseudomonas aeruginosa* and a group consisting of five species that are not separated from each other and that all belong to the Enterobacteriaceae family (*Escherichia coli*, *Citrobacter koseri*, *Klebsiella pneumoniae*, *Serratia marcescens*, and *Proteus mirabilis*). To observe the performance of different algorithms, the MS dataset consisted of 135 samples and 185,001 *m*/*z* ratios that can be replaced by a reduced dataset of peak-picked variables with 135 samples and 1964 variables. Fig. 5 provides the NMF biclustering on this peak-picked dataset. If we reconstruct the selected bicluster, we will identify the samples and variables that are both associated in this bicluster.

Fig. 4B provides the overall prediction result on this MS dataset. It indicates that NMF achieves the highest bicluster match score on the metabolic data among the various methods. Moreover, NMF has the fastest speed among the various algorithms (Fig. 4C).

With a special focus on the thresholding algorithms, OTSU [26], fuzzy c-means (FCM) [27], iterative selection (Iter) [28] and maxime entropy (MaxEn) [26] are utilized for finding the bicluster from the score and loading generated by the matrix factorization methods. We compared the bicluster match score of these thresholding methods on our synthetic datasets and the bacteria MS (peak-picking) dataset. As shown in Table 3, the OTSU and Iter perform better than FCM and MaxEn. The performance of OTSU/Iter thresholding is still higher despite sparseness on the spectroscopic data, which validates our method (NMF with thresholding) as superior in recognizing the bicluster than SNMF.

**Table 3:** The bicluster match score of matrix factorization by thresholding methods on the synthetic data1 (noise level δ = 0.25), synthetic data 2 and bacteria MS peak-pick dataset.

	PCA			NMF		
Thresholding	Synthetic data1	Synthetic data2	Bacteria MS data	Synthetic data1	Synthetic data2	Bacteria MS data
OTSU	0.72	0.61	0.36	0.91	0.79	0.99
FCM	0.65	0.53	0.44	0.62	0.61	0.87
Iter	0.72	0.61	0.28	0.85	0.77	0.99
MaxEn	0.57	0.59	0.23	0.61	0.60	0.47

The biclustering method is more useful on the MS dataset with sharp peaks and big signal-to-noise ratio than NMR data. Here, the bacterial dataset contains hundreds of unique spectral features with the signal to noise ratios (SNR) in the order of 10,000 times. High correlation between discriminating signals in each member of the same group. Moreover, the bacterial dataset has a hierarchical structure with the difference between Gram positive and Gramnegative bacteria (family level), representing where most the variance in the global data lies. With NMF (as with k-means), the number of output vectors (factors) is predefined, so the hierarchical nature is bypassed.

## 4 Conclusions

Spectroscopic data commonly contains around a thousand peaks from possibly hundreds of metabolites, is widely used in metabolomics to provide information on metabolite profiles of complex biological mixtures. This work represents a matrix factorization based biclustering model of spectroscopic data. In this paper, we use a novel bi-cross validation to decide the number of factors in the spectroscopic data matrix factorization tools. The simple thresholding methods (e.g., NMF) are used to transfer the spectroscopic data into two matrices. In this paper, we make the comparison among the various bi-clustering schemes on the simulation dataset, and the conclusion is that the simple matrix factorization tool is superior. Moreover, we develop a visualization graphical user interface and work on the bacterial dataset examples to test our biclustering model. The results demonstrate that the proposed matrix factorization based method for biclustering is useful for spectroscopic data. The future work would include the application of the proposed biclustering method on other biological datasets such as gene expression datasets.
