# Supplementary material for: Bi-clustering of metabolic data using matrix factorization tools
Source: Methods. 2018 Dec 1;151:12–20. doi: 10.1016/j.ymeth.2018.02.004 (PMC6297113; doi:10.1016/j.ymeth.2018.02.004)
Supplement: Supplementary data 1 [file mmc1.docx]

**
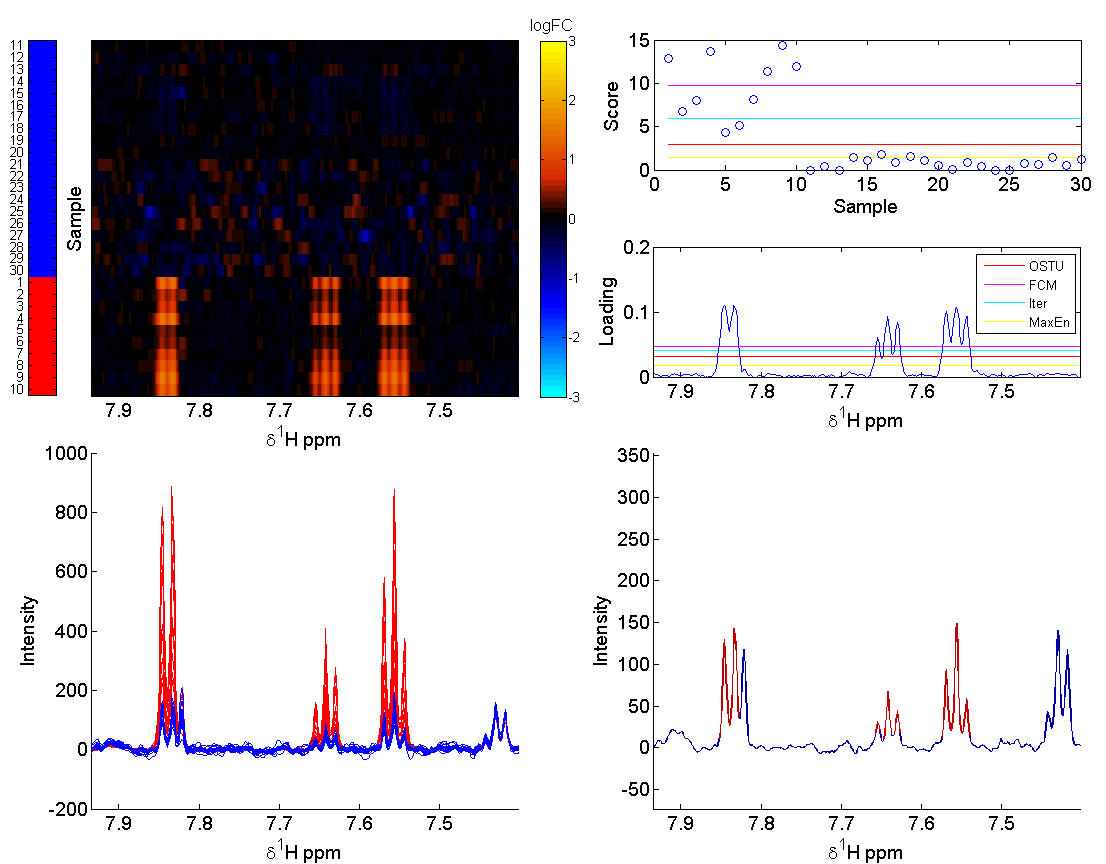
**

**Figure S1. The biclustering of synthetic dataset 2 using NMF: The left-bottom and right-bottom subplot separately shows the ^1^H-NMR spectra and the median of the spectra generated from MetAssimulo. The samples(left-bottom) and variables(right-bottom) within the selected bicluster are separately marked with red. The left-top section of the plot indicates the log transform of fold change according to mean of the ^1^H-NMR spectra. The samples within the selected bicluster are marked with the red in the left colour bar. The right-top section of the plot represents the score and loading of the dataset and the comparison of thresholding algorithms: OTSU(red), FCM(magenta), Iter(cyan) and MaxEn(yellow). The horizontal axis of the subplot (i.e.** [**chemical shifts**](http://en.wikipedia.org/wiki/Proton_NMR#Chemical_shifts)**: ^1^H ppm) keeps in consistent with each other when selecting the compounds.**
